# Indirect Exposure to Atrocities and Post-Traumatic Stress Disorder Symptoms among Aid Workers: Hemispheric Lateralization Matters

**DOI:** 10.3390/jcm13082373

**Published:** 2024-04-18

**Authors:** Einav Levy, Daniela Herzog, Chen Hanna Ryder, Rachel Grunstein, Yori Gidron

**Affiliations:** 1 Department of Social Work, Tel Hai College, Qiryat Shemona 1220800, Israel; racheligrun997@gmail.com; 2 Research Center for Innovation in Social Work, Tel Hai College, Qiryat Shemona 1220800, Israel; 3 The Israeli School of Humanitarian Action, Tel Aviv 4632825, Israel; 4 Maccabi Healthcare Services, Tel Aviv 6812509, Israel; danna.herzog@gmail.com; 5 Brain & Behavior Research Institute, Western Galilee Academic College, Akko 2412101, Israel; chenr@wgalil.ac.il; 6 Department of Nursing, Haifa University, Haifa 3498838, Israel; ygidron@univ.haifa.ac.il

**Keywords:** hemispheric lateralization, aid workers, atrocities, PTSD, moderations

## Abstract

**Background**: Humanitarian aid workers (HAWs) are indirectly exposed to atrocities relating to people of concern (POC). This may result in a risk of secondary traumatization demonstrated by post-traumatic stress symptoms (PTSSs). Previous studies have demonstrated that hemispheric lateralization (HL) moderates the relationship between threat exposure and post-traumatic stress symptoms (PTSSs). **Aims**: We hypothesized that indirect exposure to atrocities (IETA) would be positively correlated with PTSSs among HAWs with right and not left HL. **Method**: Fifty-four HAWs from several countries that provided humanitarian support in Greece and Colombia participated in this correlational and cross-sectional observation study. They completed scales relating to IETA, PTSSs were assessed using a brief, valid scale, and HL was measured. **Results**: IETA was positively and significantly related to PTSSs (r = 0.39, *p* < 0.005). Considering HL, IETA was unrelated to PTSSs among people with right HL (r = 0.29, *p* = 0.14), while IETA was related to PTSSs among people with left HL (r = 0.52, *p* = 0.008). Right HL emerged as a protective factor in the relationship between IETA and PTSS. **Conclusions**: An assessment of dominant HL can serve as one consideration among others when deploying HAWs in specific locations and roles, vis à vis IETA. Moreover, those found to have a higher risk for PTSSs based on their HL could be monitored more closely to prevent adverse reactions to IETA.

## 1. Introduction

Humanitarian settings, which entail responding to atrocities and disasters, often expose humanitarian aid workers (HAWs) to the risk of injuries, assault, kidnapping, robbery, the hostility of local populations, or other threats [1,2]. These threats can also come along with witnessing or hearing others’ stories of suffering. This exposure can lead to secondary traumatic stress (STS), which is defined as “the stress deriving from helping others who are suffering or who have been traumatized” [3]. These various stressors, which may include STS, vicarious traumatization, and compassion fatigue [4], might lead to post-traumatic stress disorder (PTSD) and a sense of burnout among HAWs in the long run.

Indeed, in a systematic review of fourteen studies, the prevalence of mental health problems, including PTSD, among HAWs was similar to or higher than various controls [5]. Another systematic review and two meta-analyses of 15 studies found moderate to severe STS among HAWs working with POC [6]. More specifically, in a study focusing on 376 HAWs in Uganda, 68% were depressed, and 53% were anxious [7]. In another study focusing on 267 HAWs following an earthquake in Pakistan, the prevalence of PTSD was 42.6% [8]. Other studies found a prevalence of 40% among Singaporean relief teams, with symptoms of acute stress reaction (ASR) [9], and 24.2% of Turkish Red Crescent team members showed PTSD symptoms [10]. Both studies were conducted following the 2004 tsunami in Southeast Asia. The same pattern was identified in a study focusing on 446 HAWs from China, where 30% developed PTSD [11]. Other symptoms related to trauma were found in various studies, such as burnout among 40% of HAWs from 44 different countries [12], and alcohol abuse was found among 16.2% of HAWs in Kosovo [13].

Risk factors for such stress responses include exposure to extremely stressful events such as dealing with dead bodies [14], previous exposure to disaster [10], length of mission [15], and low socio-economic status [16], among other factors. Protective factors, on the other hand, included early experience and training [17], perceived organizational support [18], tolerance for ambiguity [19], coping strategies, emotional intelligence [20], and serving as non-local staff [13].

The studies reviewed above suggest that not all HAWs develop PTSD despite direct or indirect exposure to atrocities (IETA). Are there protective factors in this context? Reviewing the nature of both risk and protective factors shows that these factors can be grouped into demographic background (age and socio-economic status), social environment (support of the organization and support of family and friends), or characteristics of a specific humanitarian mission (length of the mission, being local, or past exposure).

Factors that, to the best of our knowledge, have not yet been explored in a humanitarian context are neuro-psychological factors. One emerging neuro-psychological moderating protective factor is hemispheric lateralization (HL). This reflects the general tendencies to activate or use functions of one hemisphere more than the other, e.g., [21]. 

Briefly, the two hemispheres modulate immunological and psychological responses differently. The left hemisphere is involved in analytic, local thinking, positive affect, anger, behavioral activation and the approach system, and high cellular immune system functioning. Conversely, the right hemisphere is involved in intuitive, parallel and global perception, negative affect (anxiety and depression), behavioral inhibition and the avoidance system, and poor cellular immunity [22,23,24]. In humans, the side of the dominant hand is weakly related to HL. This was supported in a study of 69 French students using the line bisection test detailed below [25].

Several methods for measuring HL exist. The gold standard measure of HL includes EEG and neuroimaging techniques [26,27]. In those tests, brain activity is measured at rest and when exposed to visual or emotional stimuli. In these tests, one could measure the delta of activity levels between the same regions on the left and right hemispheres. Another measure includes the dichotic listening test, which examines the ability to process auditory information in one ear while masking information provided to the other ear. While this method has been used for decades in research, its reliability and validity are questionable [28]. While these measures are direct measures of HL and brain activity, they require a lab, equipment (e.g., neuroimaging), or a sterile and quiet environment (dichotic listening). A simpler measure suitable to humanitarian field work is the line bisection test. One type of this test is the landmark test, where participants are asked to judge which side is longer in a bisected line [29]. Another type is the line bisection test, where participants receive a series of non-bisected horizontal lines, in which they need to bisect in the middle [30]. In both types, left-side bias reflects RHL and vice versa. 

These behavioral and emotional differences mediated by the hemispheres are synthesized within a broader theoretical framework, which we now present.

### The Approach-Withdrawal Theory

Gray and McNaughton [31] proposed a behavioral activation system (BAS) and a behavioral inhibition system (BIS) in their reinforcement sensitivity theory. The BAS responds to stimuli of reward and non-punishment, elicits positive emotions, and leads to approach behavior and active avoidance. The BIS responds to the stimuli of punishment and non-reward, as well as to novel stimuli and innate fear-eliciting stimuli, wherein it elicits the affective state of anxiety and leads to behavioral inhibition and passive avoidance. Hofman’s [23] review helps to integrate the diverse and, at times, inconsistent, findings into a more coherent framework. He concluded that the right hemisphere represents predominantly the BIS and withdrawal emotions (anxiety and depression). In contrast, the left hemisphere represents the BAS, which reflects approach emotions and behaviors (positive affect, hostility, and impulsiveness).

The psychological differences controlled by the two hemispheres are also related to the impact of stressors on wellbeing. In a series of studies, exposure to stressors was positively correlated with poor mental health only among people with right HL. Furthermore, an experimental stressor increased perceived stress only among those with right but not with left HL [32]. In a following study, the perceived extent of exposure to missiles was positively correlated with PTSD levels among Israelis with right but not left HL [33].

There are inconsistencies in the literature concerning right versus left brain activity and PTSD. Studies associating LHL show that greater right alpha brain asymmetry (reflecting greater left brain activity) is related to PTSD [34]. In contrast, greater right-side activity in the amygdala, a region that processes threat, was related to exposure to trauma in a meta-analysis [35]. The picture may be more complex, such that certain PTSD symptoms, e.g., intrusive thoughts resulting from verbal processing, may be associated with LHL, while pictorial intrusions and arousal might be associated with RHL. Finally, it may be context-dependent and subject to direct versus indirect exposure to traumatic events.

To the best of our knowledge, the moderating role of HL among HAW has never been examined. The purpose of this study was to explore the moderating role of HL in the relationship between indirect exposure to atrocities and PTSD among HAWs. In line with past studies [32,33], we hypothesized that a positive correlation between IETA and PTSD would be found only among aid workers with right HL.

## 2. Method

### 2.1. Participants

The sample was recruited by the principal investigator in a humanitarian context and by approaching HAWs of two international humanitarian agencies who were working with the investigator. Those who had worked for at least a year in Greece and Colombia participated. The first agency is NATAN, an Israeli nongovernmental organization (NGO), and the second organization is Cadena, a Mexican NGO. The nature of the humanitarian work was mental health and psychosocial support, the distribution of non-food items, and legal aid provided to forced migrants due to internal conflicts in their country of origin.

In Greece, the HAWs were working with forced migrants from countries in North Africa or the Middle East. These forced migrants fled their homeland due to internal conflict and threats to their lives due to civil unrest. In Colombia, the forced migrants originated from Venezuela, where civil unrest is taking place due to socio-economic and political adversities.

Fifty-four participants took part in the study; all were above 18 years old and proficient in English. All of the participants gave their consent to participate in this study, which was approved by the ethics committee of Tel Hai academic college.

### 2.2. Procedure

After providing their informed consent, the participants completed scales assessing background information, an IETA scale, and a PTSD scale. HL was measured via a neuropsychological test (see below).

### 2.3. Measures

Sociodemographic and background variables: These included age, gender, country of origin, profession, number of past humanitarian missions, number of days in the mission that they participated in while completing the scales of this study, number of people of concern (POC) that the HAWs were exposed to, position in the team (member or management), and their dominant hand.

Level of indirect exposure to atrocities (IETA): This was assessed using a scale of six items that were developed specifically for this study (see Table 1). Each item was rated on a 1–5 scale, reflecting the number of POC, where 1 = none, 2 = 1–10, 3 = 11–20, 4 = 21–50, and 5 = more than 50 POCs to which the HAWs were exposed to.

These items reflected exposure to reports of physical or mental abuse by adults and children. In the present study, the internal reliability of the scale was high (Cronbach alpha = 0.89). The scores across the six items were summed to reflect the level of IETA. Those levels were positively correlated with the number of POC HAWs worked with (r = 0.65, *p* < 0.001).

PTSD symptoms: These were assessed using a validated seven-item scale of Yes/ No options [36]. Each item referred to a different symptom of PTSD. For instance, one item was, “Did you avoid being reminded of this experience by staying away from certain places, people, or activities?”. Another example is “Did you begin to feel that there was no point in planning for the future?”. A higher sum reflects a higher probability of PTSD. In this study, the internal reliability was low (Cronbach alpha = 0.54).

Hemispheric Lateralization: This was measured using the line bisection test, which was validated against an EEG measure [30]. Additionally, greater left-side biases in this test (reflecting RHL) were found among anxious patients [37]. This test included fourteen horizontal lines, in which the participants had to indicate, with a vertical line, the perceived center of each of the 14 lines. Deviating to the right of the real middle reflects LHL (scored with positive values), while deviating to the left of the real middle reflects RHL (scored with negative values). Participants’ deviations were averaged across the 14 lines. A higher score reflected LHL. The internal reliability was adequate (Cronbach alpha= 0.81).

### 2.4. Statistical and Data Analyses

These analyses included descriptive statistics and inferential statistics. The latter included Pearson correlations between the IETA scale and PTSD in the full sample and then in the LHL and RHL subsamples (split at the median HL score) separately. This was repeated with partial correlations controlling for relevant confounders.

To test the interaction effect of HL X exposure, we used hierarchical multivariate regression. We tested whether the interaction of HL X exposure added to explaining the variance in PTSD beyond the effects of exposure and HL’s main effects alone.

All of the statistical analyses were performed using SPSS 27.

## 3. Results

Table 2 depicts the characteristics of the sample. The majority of participants came from Greece (27.6%) and the Netherlands (17.2%), and the rest came from various other countries. The mean age of the participants was just over 31 and ranged between 19 and 65 years. The mean number of humanitarian missions per individual HAW was just below six. Sixty percent had a clinical profession (physicians, nurses, etc.), and the majority were women.

In the *t*-test, there was no significant difference between left- and right-handed participants in terms of HL (t(81) = 0.77, *p* = 0.44).

In the full sample, exposure to atrocities was positively and significantly related to PTSD (r = 0.39, *p* < 0.005). We conducted a further exploratory analysis to examine which specific atrocity item correlated with PTSD. In this analysis, the scores of the first two items (hearing witness statements from POC about atrocities) were unrelated to PTSD, while the scores of the other four items (directly seeing adults or children POC who had experienced physical/mental abuses) were positively and significantly correlated with PTSD (see Table 2).

In hierarchical multivariate regression, the interaction of HL X exposure added a non-significant 1.1% to the variance in PTSD after controlling for the effects of exposure and HL’s main effects alone (F-change (1.79) = 0.86, *p* = 0.36). Even though this interaction was not significant, we nevertheless apriori conceptualized HL as a dichotomized variable—left versus right HL.

We then classified the participants according to the median HL. Importantly, there were no significant differences in terms of the extent of exposure to atrocities (t(51) = 0.4, *p* > 0.05) and PTSD scores (t(51) = 1.3, *p* > 0.05) between those with LHL and RHL.

The extent of exposure to atrocities was unrelated to PTSD among people with RHL (*n* = 27, r = 0.29, *p* = 0.14), while extent of exposure to atrocities was related to PTSD among people with LHL (*n* = 25, r = 0.52, *p* = 0.008). When controlling for the effects of age, gender and profession, exposure to atrocities was still unrelated to PTSD among people with RHL (r = 0.24, *p* = 0.26), while exposure to atrocities remained related to PTSD among people with LHL (r = 0.51, *p* = 0.03).

The previous analysis was based on a mathematical median HL cutoff, not necessarily reflecting a real HL. Thus, we performed another analysis by splitting the sample according to those with a mean positive deviation from the middle (LHL) and those with a mean negative deviation from the middle (RHL) to reflect HL more precisely. In this classification, approximately 70% of the sample had RHL, and approximately 30% of the sample had LHL. The exposure to atrocities only tended to be related to PTSD among people with RHL (r = 0.28, *p* = 0.09), while exposure to atrocities was significantly and strongly related to PTSD among people with LHL (r = 0.57, *p* = 0.03).

Finally, we ran several analyses to rule out additional explanations. There was no significant difference in the percentage of HAW with clinical professions between those with LHL and RHL (x^2^ (1) = 2.3, *p* > 0.05). Furthermore, none of the background variables (e.g., age, gender, profession, etc.) were related to PTSD.

## 4. Discussion

The objective of this study was to examine the relationships between the newly developed IETA experienced by POC and the PTSD symptoms of HAWs as a function of HL.

For the purpose of the present study, we developed a new Indirect Exposure to Atrocities scale. The internal reliability of the scale and its correlations with both the number of POC that the participants worked with and their PTSD level support the reliability and validity of this newly developed scale.

In the full sample, exposure to the atrocities experienced by POC was positively and significantly correlated to PTSD among HAWs. Importantly, this correlation was significant only in HAWs with LHL and not with RHL. These correlations remained intact after statistically controlling for age, gender, and profession. These findings suggest that mere exposure to humanitarian crises affects the psychological wellbeing of HAWs, especially among those with LHL.

These findings contradict our hypothesis, in which we anticipated that among HAWs with RHL there would be a positive correlation between levels of IETA and PTSD, similar to past studies among other populations [32,33]. In those studies, exposure to stressful life events or acute stress positively correlated with poor mental health in people with RHL. However, in the current study, surprisingly, these correlations were only observed among those with LHL.

The correlation between the level of IETA and PTSD, specifically in HAWs with LHL, can be explained through the lens of brain asymmetry and its impact on the indirect processing of traumatic experiences. Studies have shown that the left hemisphere is typically associated with language processing and analytical thinking, while the right hemisphere is linked to emotional processing and arousal [38]. In the context of PTSD, the verbal re-experiencing component of the disorder (intrusive thoughts), which involves the persistent activation of neural networks related to traumatic memories, may be more pronounced in individuals with left hemispheric dominance [39]. Research has indicated that individuals with left hemispheric dominance may exhibit altered functional connectivity patterns, particularly in the frontal areas, which could contribute to the manifestation of PTSD symptoms [40]. Furthermore, right alpha asymmetry (greater LHL) is associated with PTSD severity [34]. Additionally, structural abnormalities in brain regions such as the corpus callosum and the cerebellum have been associated with PTSD, suggesting that interhemispheric communication and cerebellar function play a role in the disorder [41,42]. The protective role of right hemispheric lateralization in mitigating the impact of indirect exposure to atrocities on PTSD among HAWs observed in the present study could be attributed to the right hemisphere’s involvement in visual–spatial processing and emotion regulation [43]. HAWs in a humanitarian context may process and re-experience the atrocities more verbally due to the indirect nature of the exposure. The right hemisphere may facilitate more adaptive coping mechanisms in terms of processing traumatic experiences, thereby reducing the risk of developing severe PTSD symptoms [44] when indirectly exposed to atrocities. In conclusion, the observed correlation between IETA and PTSD, specifically in individuals with LHL, among HAWs underscores the importance of considering hemispheric lateralization in understanding the psychological impact of indirect exposure to atrocities. The findings suggest that individuals with left hemispheric dominance may be more vulnerable to the development of PTSD symptoms following indirect exposure to traumatic experiences, highlighting the need for tailored interventions and support strategies based on an individual’s hemispheric dominance.

An additional explanation could be derived from the differences in terms of approach versus avoiding coping mechanisms between RHL and LHL individuals. One of the characteristics of LHL is an approach coping mechanism that includes the tendency to approach other people compared to those with RHL, who are characterized by more avoidance behavior [45,46]. Consequently, among HAWs with LHL, approach coping may result in greater attachment to the POC reporting their atrocities, with the outcome being an increased risk of PTSD during their mission. Though the right hemisphere is associated with empathy [47], a term relevant to our results, the behavioral–emotional patterns of approach coping (LHL) versus avoidance coping (RHL) may have had a more important role, thus explaining our pattern of results.

In contrast, among HAWs with RHL, avoidance behavior may distance them from the POC, hence minimizing the risk of PTSD even if exposed to atrocities. Furthermore, it is possible that the global perception, together with the avoidance of people with RHL, could be manifested in the ability to perceive their experience as part of a broader mission and humanitarian goal. This could help to minimize and put their IETA in perspective, a cognition found to regulate emotion in a meta-analysis of 306 to regulate emotion [48].

Risk factors for adverse psychological symptoms among HAWs can be grouped into pre-deployment factors, such as training and personality; peri-deployment factors, such as trauma exposure; and post-deployment factors, such as social support and media [49]. The current study focused on a peri-deployment factor of POC reported atrocities and identified a pre-deployment factor, namely HL, which moderates the effect of atrocities on PTSD symptoms.

We explored which specific items of the IETA scale correlated with PTSD. Indeed, four of the six items reflecting witnessing damage to adults or children among POC positively correlated with PTSD. In contrast, indirectly witnessing (hearing and not seeing) items were unrelated to PTSD. These results suggest that visually processing atrocities may have a greater impact on HAWs than hearing about atrocities alone. These results point to the gradation of the IETA experience. These results are in line with past studies that found differences in PTSD prevalence subject to direct vs. indirect exposure to stressful events or atrocities [50] and that indirect exposure might lead to PTSD, yet with lower probability [51].

The present study had several limitations. First, the size of the sample was relatively small. This may have masked potentially significant results should we have had a larger sample. However, a larger sample may not change the effect sizes observed in the present study. Importantly, there was no a priori sample size calculation since we did not know in advance how many people would participate from a humanitarian context. It should be noted that the relatively small sample size resulted from the nature and the context in which the study was performed—an emergency humanitarian disaster.

Second, we did not include a neurological measure of HL, such as EEG. Furthermore, factors influencing resilience and stress, such as familiarity with peers or pre-deployment training, were not explored in this study. In addition, secondary traumatization was not formally assessed with a suitable instrument. Instead, we assessed PTSSs because we anticipated a more direct reaction in HAWs when viewing actual physical manifestations of atrocities relating to POC. Finally, this was a cross-sectional study; therefore, no directionality of association can be inferred. HL has state and trait characteristics; therefore, it is possible that, due to the IETA, participants’ HL may have shifted to the right side [52]. Future studies should measure HL several times, including during pre-deployment, as a baseline. These results need to be replicated in a larger sample using neurological measures and a prospective design together with an assessment of secondary traumatization.

Future studies can explore not only the dominant hemisphere but also the extent of this dominance. Secondly, humanitarian agencies can measure the HL of their staff or their newly recruited workers. An assessment of the dominant HL can serve as one consideration among others in deploying HAWs in specific locations and roles, vis à vis IETA. Moreover, those found with a higher risk for PTSD based on their HL can be monitored more closely to prevent adverse reactions to IETA.

## Figures and Tables

**Table 1:** The table shows the correlations between atrocities items and PTSD.

Variable	R
How many adults have told you about their physical atrocities?	0.25
How many adults told you about their mental atrocities and abuses?	0.22
How many adults have you seen with physical injuries?	0.36 **
How many adults have you seen suffering?	0.32 *
How many children (<age 18) have you seen with injuries?	0.34 *
How many children have you seen suffering?	0.32 *

PTSD—post-traumatic stress disorder * *p* < 0.05, ** *p* < 0.01.

**Table 2:** The table shows the sample characteristics.

a. Categorical variables	
Variable	Percent
Gender	
Men	25.9%
Women	74.1%
Profession	
Technical/Logistics	40%
Clinical	60%
Dominant hand	
Right	94.4%
Left	5.6%
b. Continuous	
Variable	Mean (SD)
Age	31.58 (9.56)
Number of missions	5.86 (16.19)
Hemispheric lateralization	−0.19 (0.41)
Exposure	18.82 (6.93)
Perceived threat	1.96 (0.96)
PTSD	1.06 (1.25)

PTSD—post-traumatic stress disorder.

## Data Availability

If required, our data can be submitted.

## Abbreviations

ASR	Acute stress reaction
HAW	Humanitarian aid workers
IETA	Indirect exposure to atrocities
LHL	Left hemispheric lateralization
RHL	Right hemispheric lateralization
NG0	Nongovernmental organization
POC	People of concern
PTSD	Post-traumatic stress disorder
PTSS	Post-traumatic stress symptoms
STS	Secondary traumatic stress
